# An endocannabinoid receptor polymorphism modulates affective processing under stress

**DOI:** 10.1093/scan/nsy083

**Published:** 2018-10-02

**Authors:** Lisa Wirz, Martin Reuter, Andrea Felten, Lars Schwabe

**Affiliations:** 1 Department of Cognitive Psychology, University of Hamburg, Hamburg Germany; 2 Department of Differential and Biological Psychology, University of Bonn, Bonn, Germany

**Keywords:** endocannabinoids (eCBs), stress, medial prefrontal cortex, affective processing, emotional memory

## Abstract

Stress has a critical impact on affective and cognitive processing. Based on rodent data suggesting that endocannabinoid signaling via CB1 receptors serves as an emotional buffer, we hypothesized that a common variant of the gene coding for the CB1 receptor modulates affective processing under stress (*CNR1*; rs1049353 A *vs* G allele). Therefore, 139 participants, genotyped for this polymorphism, underwent a stress or control manipulation before they viewed emotionally neutral and negative pictures in a magnetic resonance imaging scanner. The ventromedial prefrontal cortex, known for its crucial role in emotion regulation, was significantly more activated in AA/AG *vs* GG genotype carriers when viewing negative pictures after stress. Under no-stress conditions, AA/AG genotype carriers showed enhanced crosstalk between the ventrolateral prefrontal cortex and the amygdala. We further assessed participants’ 24 h-delayed memory for the presented pictures and found that memory performance correlated with amygdala and hippocampus activity and connectivity in stressed carriers of the AA/AG but not the GG genotype. These findings underline the modulatory role of the endocannabinoid system in stress effects on emotion and cognition and provide insights into the neural mechanisms that may contribute to the suggested protective effect of the AA/AG genotype of the CB1 receptor polymorphism against stress-related psychopathologies.

## Introduction

Stressful events have a major impact on mental health and may contribute to psychopathologies such as addiction, post-traumatic stress disorder (PTSD) or major depression (McEwen, 2004; de Kloet *et al.*, 2005; Chrousos, 2009). These psychopathologies are at least partly driven by stress-induced changes in affective processing (Karl *et al.*, 2006; Leppänen, 2006). Indeed, there is strong evidence that stress alters our responses to emotional stimuli (Ellenbogen *et al.*, 2002; van Stegeren *et al.*, 2005; Fox *et al.*, 2010; Weymar *et al.*, 2012). These changes in affective processing have mainly been attributed to the effects of stress-induced increases in catecholamines and glucocorticoids on the amygdala, prefrontal cortex (PFC) and hippocampus (de Kloet *et al.*, 2005; Arnsten, 2009; Joels and Baram, 2009; Schwabe *et al.*, 2013), brain regions crucial for affective processing (Sergerie *et al.*, 2008; Kalisch, 2009; Etkin *et al.*, 2011). Recent findings, however, point to another important player in the effects of stress on affective processing: the endocannabinoid (eCB) system (Campolongo and Trezza, 2012; Morena *et al.*, 2016).

The eCB system is a lipid-signaling system in the brain that modulates neurotransmitter release (Kogan and Mechoulam, 2006). The system is composed of the eCB ligands anandamide and 2-arachidonoylglycerol (2-AG), the cannabinoid receptors CB1 and CB2 and the enzymes involved in eCB synthesis and metabolism [fatty acid amide hydrolase (FAAH) for anandamide and monoacylglycerol lipase for 2-AG; Mechoulam and Parker, 2013]. ECBs and CB1 receptors are abundantly present in the amygdala, hippocampus and PFC (CB2 receptors are located mainly in the periphery; McPartland *et al.*, 2007; Morena and Campolongo, 2014). In addition, eCBs are rapidly synthesized on demand and retrogradely activate CB1 receptors in these brain regions, thus putting the eCB system and in particular CB1 receptors in a prime position to modulate stress effects on affective processing. In line with this idea, rodent studies showed that eCB signaling via the CB1 receptor can regulate activation of the hypothalamus-pituitary-adrenal (HPA) axis and modulate affective processing under stress (Lutz, 2009; McLaughlin *et al.*, 2012; Wang *et al.*, 2012; Bedse *et al.*, 2014; Gray *et al.*, 2015). For instance, injection of a CB1 receptor agonist into the basolateral part of the amygdala (BLA) prevented the stress-induced glucocorticoid increase in rats (Ganon-Elazar and Akirav, 2009) and anandamide was reported to have anxiolytic effects (Lutz *et al.*, 2015). Furthermore, administration of a CB1 receptor antagonist into the medial PFC (mPFC) prolonged the corticosterone response to a stressor, suggesting that termination of HPA axis activation by glucocorticoids within the mPFC critically depends on eCB signaling via the CB1 receptor (Hill *et al.*, 2011). Thus, the eCB system has been suggested to act as an emotional buffer system that is crucial for appropriate affective responding (Lutz, 2009; Campolongo and Trezza, 2012; McLaughlin *et al.*, 2014; Lutz *et al.*, 2015).

So far, experimental evidence for the role of the eCB system in affective processing under stress comes almost exclusively from animal studies. There is, however, first evidence that at least some of the animal findings can be translated to humans. In particular, clinical studies tested the effects of the CB1 receptor antagonist rimonabant and showed that it led to decreased activity of brain reward regions in response to pleasant stimuli (Horder *et al.*, 2010) and to a negative bias in memory recall (Horder *et al.*, 2009; Horder *et al.*, 2012). However, because rimonabant administration also led to significant increases in anxiety and depressive mood (Mitchell and Morris, 2007; Hill and Gorzalka, 2009; Goodwin *et al.*, 2012), which further underlines the relevance of CB1 receptors in affective processing, it had to be taken off the market and pharmacological manipulations of the eCB system are thus not feasible in humans anymore. Furthermore, studies on the effects of cannabis and CB1 receptor agonists, as is generally the case in pharmacological studies, may be less informative in this context, because the effects of exogenous cannabinoids may be substantially different since they lack the spatial and temporal specificity of endogenous eCBs (Steiner and Wotjak, 2008; Akirav, 2011, 2013). An alternative strategy to target the function of eCBs in humans, however, is an imaging genetics approach, employing the individual genetic variance in eCB activity. Single nucleotide polymorphisms (SNPs) in the CB1 receptor gene (*CNR1*) have been linked to mood and anxiety disorders, such as PTSD and major depression (Hillard *et al.*, 2012). In particular, carrying at least one copy of the minor A allele of the exonic rs1049353 polymorphism has been proposed as a protective factor that reduces the risk of depression after stressful events (Agrawal *et al.*, 2012), whereas homozygous carriers of the major G allele were found to be at higher risk for antidepressant treatment resistance (Domschke *et al.*, 2008). While these findings suggest that a genetic variant of the CB1 receptor may be linked to stress-related psychopathologies, how eCBs and in particular this CB1 receptor polymorphism (rs1049353) may alter affective processing in humans under stress is completely unknown.

The primary aim of the present study was therefore to determine if and how a genetic variant of the CB1 receptor gene (rs1049353) modulates the neural processing of affective information after stress. For this purpose, healthy participants were genotyped for the rs1049353 polymorphism and randomly assigned to a stress [Trier social stress test (TSST); Kirschbaum *et al.*, 1993] or control manipulation. Following the experimental manipulation, participants were presented with emotionally negative and neutral pictures, while their brain activity was measured using functional magnetic resonance imaging (fMRI). We hypothesized that the CB1 receptor polymorphism (rs1049353) would modulate the stress effects on activity in brain regions that are crucial for affective processing, such as the amygdala and the mPFC. In particular, we predicted that in response to the stress manipulation, AA/AG compared to GG genotype carriers would show reduced amygdala activity and increased activity in prefrontal areas that are implicated in emotion regulation (Urry *et al.*, 2006; Banks *et al.*, 2007). Although this study focused mainly on the modulatory role of the rs1049353 genotype in affective processing after stress, we were also interested in potential effects of this polymorphism on subsequent memory for the neutral and emotional stimuli, because eCBs are also thought to play a crucial role in the emotional modulation of memory (Campolongo *et al.*, 2009; Atsak *et al.*, 2015) and emotional memory processes are highly relevant in stress-related psychopathologies such as PTSD (Pitman *et al.*, 2012; de Quervain *et al.*, 2017). Therefore, participants additionally completed free recall and recognition tests for the presented pictures 24 h after encoding. In terms of the modulation of emotional memory processes under stress, we expected that the CB1 receptor polymorphism might modulate the activity and interplay of the amygdala and hippocampus, the two key regions in emotional memory formation (Cahill and McGaugh, 1998; McGaugh, 2004; Roozendaal *et al.*, 2009).

## Materials and Methods

### Participants and experimental design

In total, 139 young, healthy, normal-weight Caucasian volunteers [67 women; mean age = 23.4 years, *s.d.* = 3.5 years; mean body mass index (BMI) = 22.51 kg/m^2^, *s.d.* = 2.27] from European ancestry participated in this experiment. Exclusion criteria, assessed by means of a standardized interview, included medication or drug intake (including cannabis consumption), any past or current neurological or psychiatric disorders, smoking, a BMI < 18 kg/m^2^ or > 26 kg/m^2^, as well as any contraindications to fMRI measurements. In addition, women were not tested during their menses. The experiment was approved by the ethical review board of the German Psychological Society (reference: LS072014) and the local ethics committee at the University of Bonn and is in accordance with the Declaration of Helsinki. This experiment is part of a larger study on genotype-dependent differences in cognitive processes under stress (Wirz *et al.*, 2017a; Wirz *et al.*, 2017b).

A 2 × 2 × 2 mixed design with the between-subjects factors treatment (stress *vs* control manipulation) and *CNR1* genotype (AA/AG *vs* GG genotype carriers) and the within-subject factor emotionality (negative *vs* neutral) was used to investigate *CNR1* genotype-dependent differences in stress effects on neural processing of affective information. Participants were randomly assigned to a stress or control condition. Technical difficulties and excessive head motion in the magnetic resonance imaging (MRI) scanner led to the exclusion of two participants. Additionally, three participants had to be excluded due to missing picture ratings or recall data, leading to a final sample of 134 participants (stress: 34 males, 33 females; control: 36 males, 31 females). For the genetic analyses, another three participants had to be excluded due to missing data for the *CNR1* SNP of interest (131 participants; stress-AA/AG genotype: 16 males, 17 females, stress-GG genotype: 18 males, 15 females; control-AA/AG genotype: 19 males, 8 females, control-GG genotype: 17 males, 21 females).

### Genetic analyses

Participants were genotyped for the rs1049353 SNP of the *CNR1* gene on chromosome 6q14-q15. This SNP is located on the coding exon of *CNR1* and has been associated with depression in response to stress exposure as well as with PTSD (Hill and Patel, 2013; Mota *et al.*, 2015). For genetic analysis, DNA was extracted from buccal cells. Automated purification of genomic DNA was conducted by means of the MagNA Pure^®^ LC system using a commercial extraction kit (MagNA Pure LC DNA isolation kit; Roche Diagnostics, Mannheim, Germany). Genotyping of the *CNR1* polymorphisms was performed by MALDI-TOF mass spectrometry using the iPLEX assay and the Sequenom MassARRAY platform. For all further analyses, homo- and heterozygous carriers of the rs1049353 minor A allele (AA/AG genotype carriers), which seems to be protective against the effects of stress (Hill and Patel, 2013), were treated as one group and tested against homozygous carriers of the major G allele (GG genotype carriers).

### Stress and control manipulation

In the stress condition, participants underwent the TSST, a commonly used laboratory stressor that has been shown to induce a reliable increase in autonomic nervous system and HPA axis activity (Kirschbaum *et al.*, 1993). In a mock job interview, participants were introduced to a reserved and non-reinforcing panel that evaluated participants’ performance on two tasks. The first task consisted of a 5 min free speech about why he or she is the ideal candidate for a job tailored to his or her interests, whereas in the second task the participant was asked to count backwards from 2043 in steps of 17 for another 5 min. During both tasks, participants were videotaped. In the control condition, the participant was alone in the room, without video recordings, talked about a self-chosen topic and performed an easy calculation task (counting forward in steps of 15).

The effectiveness of the stress induction was assessed by means of questionnaires, blood pressure measurements and saliva samples. Changes in subjective mood were evaluated with a German mood questionnaire (MDBF; subscales: depressed *vs* elevated, restless *vs* calm, sleepy *vs* awake; high scores indicate elevated mood, calmness and wakefulness; Steyer *et al.*, 1994) and an additional questionnaire in which participants rated on a scale from 0 (`not at all’) to 100 (`very much’) how difficult, unpleasant and stressful they had experienced the stress or control manipulation. Blood pressure was measured using a Dinamap system (Critikon, Tampa, USA) before (−25 min), during (+10 min) and at several time points after the experimental treatment (+20 min, +90 min). To assess the HPA axis response to the TSST and control manipulation, saliva samples were collected before (−25 min) and after (+20 min, +30 min, +90 min) the experimental treatment using Salivette® collection devices (Sarstedt, Nümbrecht, Germany). Saliva samples were stored at −18°C until the end of the experiment, when the free fraction of cortisol was determined using chemiluminescence immunoassays (IBL, Hamburg, Germany).

### Affective picture task

In order to investigate *CNR1* genotype-dependent differences in the neural processing of emotional information under stress, participants viewed negative and neutral pictures while fMRI was recorded. Pictures were taken from the International Affective Picture System (IAPS; Lang *et al.*, 2008) and an in-house database which includes pictures depicting scenes with a more contemporary relevance (e.g. pictures of refugees). On the basis of previous valence ratings on a scale from 0 (`negative’) over 50 (`neutral’) to 100 (`positive’), the pictures were categorized as emotionally negative (24.19 ± 7.38) and emotionally neutral (55 ± 7.42), with 25 pictures in each emotionality category. These previous ratings (0 = `not arousing’, 100 = `very arousing’) showed that mean arousal levels were significantly larger for negative (50.32 ± 8.93) compared to neutral pictures (11.35 ± 2.84; t_(18)_ = 18.78, *P* < 0.001). In the current study, the pictures were presented for 2.5 s in the middle of the screen in a quasi-randomized order, ensuring that no more than two pictures of the same emotionality were seen one after another. Participants were asked to rate the pictures on a four-point scale [`negative’ (i), `rather negative’ (ii), `rather neutral’ (iii), `neutral’ (iv)], but they were not explicitly instructed to memorize the pictures for a subsequent memory test. Between pictures, there were fixation periods of 6–10 s (mean = 7 s), resulting in a total task duration of 8 min.

### Experimental procedure

All testing took place in the afternoon to control for the diurnal rhythm of cortisol. After participants had given written informed consent, buccal cells were collected for later genetic analyses. Participants then underwent the stress or control manipulation before they were placed inside the MRI scanner. Approximately 50 min after the onset of the stress or control manipulation, participants performed the affective picture task. At the end of the experiment, participants received a moderate monetary compensation (35 €). Although this experiment focused mainly on the modulatory effect of a CB1 receptor polymorphism (rs1049353) on the influence of stress on the neural processing of affective material, we aimed also to assess potential effects on emotional memory formation. To this end, participants were called 24 h after the affective picture task and asked to describe as many pictures as possible in as much detail as possible, so that the experimenter knew for sure to which picture the participant was referring to. When more than 60 s had elapsed after the last picture was recalled, a link to a forced choice recognition test was sent to the participants which they completed immediately after the free recall test. In the recognition test, participants saw all pictures they had seen the day before, as well as 25 negative and 25 neutral pictures that were not presented before in a randomized order. Participants indicated by button press whether they thought it was an old or a new picture and additionally specified whether they were `very sure’, `rather sure’, `rather unsure’ or `sure’ in their decision.

### Behavioral and physiological data analyses

Physiological, subjective and behavioral parameters were analyzed using mixed-design analyses of variance (ANOVAs) with time as within-subject factor and treatment (stress *vs* control manipulation) as well as *CNR1* genotype (AA/AG *vs* GG genotype carriers) as between-subjects factors. For the analyses of picture ratings, certainty ratings, free recall and recognition performance, we added emotionality (negative *vs* neutral) as within-subject factor. For our memory analyses, we focused on the number of correctly recalled pictures in the free recall test as well as hits and false alarms in the recognition test. In addition, the sensitivity index d-prime (d′) was calculated for negative and neutral pictures, using hits and false alarms according to signal detection theory (Wickens, 2002), because this measure corrects for individual response biases. Statistical analyses were performed using SPSS Statistics 22 (IBM, Armonk, USA). All reported *P*-values are two tailed and in case of violation of the sphericity assumption, Greenhouse–Geisser correction was applied. To correct for multiple comparisons in our four experimental groups, we report significant effects at a corrected α-threshold of *P* < 0.0125 (α = 0.05 / number of correlations calculated [4]).

### MRI acquisition and analyses

fMRI measurements were acquired using a 3T Trio Scanner (Siemens, München, Germany) with a 32-channel head coil. BOLD T2-weighted echoplanar functional images parallel to the anterior commissure–posterior commissure plane (37 transversal slices; repetition time (TR) = 2000 ms; echo time (TE) = 30 ms; ascending acquisition; effective voxel size = 3 × 3× 3 mm) and a high-resolution T1-weighted anatomical image (208 sagittal slices, TR = 1660 ms, TE = 2.54 ms, voxel size = 0.8 × 0.8 × 0.8 mm) were acquired.

fMRI pre-processing and data analyses using general linear modeling were performed using the SPM12 Matlab toolbox (Wellcome Trust Centre for Neuroimaging, London, UK). Functional data were slice-time and head-motion corrected as well as coregistered to the structural image using rigid-body transformations. The T1-weighted image was segmented into gray and white matter, cerebrospinal fluid, bone, soft tissue and air. Using forward deformation fields, the functional and structural scans were spatially normalized to the Montreal Neurological Institute standard brain. Finally, an 8 mm full width half maximum Gaussian kernel was used to smooth the normalized functional images.

Negative and neutral picture trials were modeled using canonical hemodynamic response functions. Fixation, button press and the six movement parameters were included as regressors of no interest. A temporal high-pass filter with a 128 s cutoff was used and contrast images were generated for negative minus neutral picture trials. These difference contrasts were then entered into second-level (group) analyses using a full factorial model with the factors treatment (stress *vs* control manipulation) and *CNR1* genotype (AA/AG *vs* GG genotype carriers). Exploratory whole-brain analyses as well as region of interest (ROI) analyses were used. A priori ROIs were corticolimbic structures known to be involved in affective processing and memory formation (i.e. the amygdala, insula and hippocampus; McGaugh, 2000; Phan *et al.*, 2002), as well as PFC areas [medial PFC (mPFC), ventromedial PFC (vmPFC), ventrolateral PFC (vlPFC)] that play a pivotal role in emotion regulation (Wager *et al.*, 2008; Motzkin *et al.*, 2015). Anatomical masks of subcortical brain regions (amygdala, insula, hippocampus) and the mPFC were taken from the Harvard-Oxford Atlas with a probability threshold of 50%, so that only voxels with a probability of at least 50% to belong to each brain region were included. Anatomical masks of the vmPFC and vlPFC were created using MARINA software (http://www.bion.de/eng/MARINA.php). For the exploratory whole-brain analyses, the significance threshold was set to *P* < 0.05 at cluster level (in a minimum of five adjacent voxels) and corrected for multiple testing [family-wise error (FWE) correction]. ROI analyses using small volume correction with an initial threshold of *P* < 0.05 uncorrected were followed by FWE correction (*P* < 0.05). Within a ROI, only clusters of at least five significant voxels are reported.

To assess group differences in the connectivity between our ROIs, we performed psychophysiological interaction (PPI) analyses. Accordingly, the first eigenvariate of the time course of our ROIs in the contrast negative minus neutral was extracted from the appropriate brain atlases and used as seed region. A general linear model with a physiological regressor (time course response in the seed region), a psychological regressor (negative minus neutral pictures) and a PPI regressor, which was calculated as the cross product of the previous two regressors, was computed. The individual PPI contrasts were then entered into second-level random-effects analyses. As these analyses reveal brain regions with a similar and task-dependent activation pattern, these regions are supposed to be functionally connected during the processing of negative *vs* neutral pictures.

To investigate whether brain activation and connectivity during picture processing were directly associated with subsequent memory performance, we correlated brain activity and functional connectivity of our ROIs to participants’ individual memory performances. For this purpose, we ran a second full factorial model and PPI models with memory performance entered as a covariate. We then extracted the contrast values of the significant clusters of voxels with MarsBarR (http://marsbar.sourceforge.net/) and correlated these with the participants’ memory performance. To compare correlations between our experimental groups, we additionally ran separate models for AA/AG and GG genotype carriers in the stress and control group, including memory performance as a covariate. Subsequently, contrast estimates were correlated with participants’ memory performance. Correlation coefficients were then transformed using the Fisher’s *r*-to-*z* transformation and the resulting *z*-scores were statistically compared.

## Results

### Genetic analyses

Genotyping participants for the rs1049353 SNP of the *CNR1* gene coding for the CB1 receptor revealed 74 (53.2%) homozygous G allele, 4 (2.9%) homozygous A allele and 58 (41.7%) heterozygous G/A allele carriers. In line with previous studies (Agrawal *et al.*, 2012; Mota *et al.*, 2015) and due to the small number of homozygous A allele carriers, homo- and heterozygous carriers were treated as one group [62 AA/AG genotype carriers (44.6%)] and tested against homozygous G allele (GG genotype) carriers. Allele frequencies (minor allele frequency = 24.26%, major allele frequency = 75.74%) were in accordance with those documented by the National Center for Biotechnology Information for Europeans and in Hardy–Weinberg equilibrium (χ^2^_(1)_ = 3.50, *P* = 0.061). GG and AA/AG genotype carriers were equally distributed in the stress (33 AA/AG genotype carriers, 38 GG genotype carriers) and control group (28 AA/AG genotype carriers, 33 GG genotype carriers; χ^2^_(1)_ = 0.77, *P* = 0.381). Given that the genotypic distribution nearly violated Hardy–Weinberg equilibrium and in light of different homozygous A allele frequencies reported in previous studies (Domschke *et al.*, 2008; Agrawal *et al.*, 2012), we reanalyzed our neuroimaging data without AA genotype carriers. Importantly, our results remained largely unchanged after excluding the AA genotype carriers (see Supplementary Material).

### Successful stress induction by the TSST

Subjective mood, as well as blood pressure and cortisol concentrations significantly changed in response to the TSST and verified the successful stress induction. Independent of *CNR1* genotype, exposure to the TSST was rated as significantly more difficult, unpleasant and stressful than the control manipulation (all *F*_(1,132)_ ≥ 71.62, all *P* < 0.001; Table 1). In addition, GG genotype carriers of the rs1049353 SNP were overall more restless than AA/AG genotype carriers (*F*_(1,130)_ = 4.92, *P* = 0.028). More importantly, however, independent of *CNR1* genotype, participants’ mood decreased and they became increasingly restless in response to the TSST compared to the control condition (time × treatment: both *F*_(2,129)_ ≥ 21.45, both *P* < 0.001; Table 1). Independent of *CNR1* genotype and treatment condition, participants became increasingly tired during the course of the experiment (time: *F*_(2,129)_ = 96.06, *P* < 0.001).

**Table 1:** Subjective stress response

	**Control**		**Stress**	
	**AA/AG genotype**	**GG genotype**	**AA/AG genotype**	**GG genotype**
**Subjective assessment**				
Stressful	24.64 ± 4.07	31.03 ± 3.36	64.12 ± 3.56	67.43 ± 3.49^***^
Difficult	25.71 ± 4.32	26.41 ± 3.30	70.88 ± 4.01	71.14 ± 3.40^***^
Unpleasant	28.93 ± 4.78	37.69 ± 3.93	66.18 ± 4.35	70.57 ± 3.50^***^
**Subjective mood**				
**Good *vs* bad mood**				
Before treatment	33.86 ± 0.71	34.45 ± 0.82	34.38 ± 0.73	34.43 ± 0.65
1 min after treatment	32.64 ± 0.82	34.15 ± 0.78	28.15 ± 1.13	27.91 ± 1.16^***^
75 min after treatment	32.14 ± 0.96	32.85 ± 0.81	30.35 ± 1.09	31.31 ± 0.82
**Calm *vs* restless**				
Before treatment	29.93 ± 0.96	33.26 ± 0.84	30.97 ± 0.98	32.60 ± 0.71
1 min after treatment	29.11 ± 1.03	31.38 ± 0.91	23.76 ± 1.15	24.79 ± 1.07^***^
75 min after treatment	30.68 ± 0.88	32.00 ± 0.98	31.59 ± 0.98	31.80 ± 0.67
**Overall calm *vs* restless**	29.90 ± 0.78	32.13 ± 0.79	28.77 ± 0.86	29.82 ± 0.58^#^
**Tired *vs* awake**				
Before treatment	29.93 ± 1.07	30.76 ± 0.98	30.03 ± 0.78	30.43 ± 0.85
1 min after treatment	28.82 ± 1.13	30.10 ± 0.99	28.82 ± 0.95	28.24 ± 0.88
75 min after treatment	22.93 ± 0.97	24.26 ± 1.00	21.38 ± 1.01	23.51 ± 0.97

Data represent means ± SEM.

Time × Treatment (stress *vs* control) ^***^P < 0.001 ^**^P < 0.01 ^*^P < 0.05

*CNR1* genotype (AA/AG *vs* GG) ^#^*P* < 0.05

**Fig. 1:**
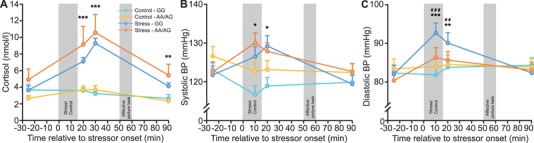
Physiological changes in the experimental groups. Independent of *CNR1* genotype and compared to a non-stressful control manipulation, exposure to the TSST led to significant increases in (**A**) salivary cortisol concentrations and (**B**) systolic blood pressure. Whereas stress also increased (**C**) diastolic blood pressure, this increase was only observed in rs1049353 GG genotype carriers. Stress *vs* Control ^***^P < 0.001 ^*^P < 0.05, Stress/GG genotype *vs* all other groups ^###^*P* < 0.001 ^#^*P* < 0.01, error bars represent SEM.

Exposure to the TSST, compared to the control condition, further led to a significant increase in cortisol concentrations (time × treatment: *F*_(3,130)_ = 17.73, *P* < 0.001; Figure 1A), which provides evidence for a stress-induced activation of the HPA axis. Stress-induced increases in salivary cortisol were not affected by *CNR1* genotype (all main and interaction effects: all *F* ≤ 0.82, all *P* ≥ 0.368). Finally, activity of the autonomic nervous system significantly increased in response to the TSST, but not after the control manipulation, as was shown by significant increases in systolic and diastolic blood pressure (time × treatment: both *F* ≥ 17.51, both *P* ≤ 0.001). Although systolic blood pressure was not influenced by *CNR1* genotype (time × genotype: *F*_(3,129)_ = 1.43, *P =* 0.236; genotype: *F*_(1,131)_ = 0.55, *P =* 0.460; Figure 1B), a time × treatment × genotype interaction for diastolic blood pressure (*F*_(3,129)_ ≥ 3.49, *P =* 0.018) showed that in rs1049353 GG genotype carriers, diastolic blood pressure increased during and immediately following the stress induction (both *F*_(1,72)_ ≥ 7.57, both *P* ≤ 0.008), whereas in AA/AG genotype carriers no such effect was observed (both *F*_(1,59)_ ≤ 1.15, both *P* ≥ 0.287; Figure 1C).

**Table 2:** Picture ratings and memory performance

	**Control**		**Stress**	
	**AA/AG genotype**	**GG genotype**	**AA/AG genotype**	**GG genotype**
**Picture ratings**				
Negative pictures	1.62 ± 0.07	1.56 ± 0.06	1.67 ± 0.08	1.58 ± 0.06^***^
Neutral pictures	3.55 ± 0.09	3.56 ± 0.06	3.55 ± 0.09	3.60 ± 0.07
**Free recall**				
Negative pictures	6.61 ± 0.53	5.95 ± 0.35	6.52 ± 0.34	6.74 ± 0.53^***^
Neutral pictures	3.07 ± 0.38	3.41 ± 0.41	3.39 ± 0.26	3.76 ± 0.38
**Recognition**				
Negative pictures hit rate	92.67 ± 1.00	91.18 ± 1.26	88.85 ± 1.54	86.94 ± 2.52^***^
Neutral pictures hit rate	85.26 ± 2.20	86.15 ± 1.89	84.85 ± 2.22	83.82 ± 2.32
Negative pictures false alarm rate	9.19 ± 1.68	9.44 ± 1.34	9.45 ± 2.49	9.88 ± 1.36^***^
Neutral pictures false alarm rate	17.56 ± 1.76	21.95 ± 2.66	19.64 ± 2.31	21.41 ± 2.75
**Certainty ratings**				
Negative pictures	3.71 ± 0.04	3.62 ± 0.04	3.62 ± 0.05	3.60 ± 0.05^***^
Neutral pictures	3.43 ± 0.06	3.40 ± 0.07	3.41 ± 0.06	3.47 ± 0.06
**D-prime**				
Negative pictures	3.04 ± 0.12	2.98 ± 0.12	2.88 ± 0.15	2.76 ± 0.14^***^
Neutral pictures	2.18 ± 0.13	2.09 ± 0.12	2.13 ± 0.13	2.04 ± 0.12

Table shows picture ratings as well as free recall and recognition performance in dependence of experimental manipulation (TSST *vs* control condition) and *CNR1* genotype (AA/AG *vs* GG). Data represent means ± SEM.

Emotionality (negative *vs* neutral) ^***^P < 0.001

**Table 3:** Significantly activated cluster peak voxels during negative picture processing

	**MNI coordinates (mm)**					
**Negative > Neutral**	**Cluster size**	***x***	***y***	***z***	***t***_**max**_	***P***_**FWE-corr**_
R post-central gyrus	1 226	51	−19	59	17.74	<0.001
R temporal inferior gyrus	917	45	−64	−7	17.51	<0.001
L medial superior frontal gyrus	1 069	−6	50	20	12.56	<0.001
L occipital inferior gyrus	1 242	−42	−76	−4	12.33	<0.001
L insula	2 053	−30	17	−16	11.59	<0.001
L fusiform gyrus	19	−30	−7	−34	6.95	<0.001
L posterior cingulate	25	0	−49	26	6.50	<0.001
L medial OFC	51	−3	44	−19	6.31	<0.001
L supramarginal gyrus	20	−66	−28	35	6.15	<0.001
R fusiform gyrus	12	30	−7	−34	5.47	<0.001
R amygdala	84	24	−1	−16	10.81	<0.001^*^
L amygdala	64	−21	−4	−16	10.68	<0.001^*^
L vmPFC	20	−6	50	11	9.24	<0.001^*^
R insula	122	33	14	−16	8.78	<0.001^*^
R vmPFC	19	0	50	−19	6.07	<0.001^*^
R vlPFC	12	54	32	8	6.01	<0.001^*^

Table shows local maxima of functional voxels (normalized voxel size = 3 × 3 × 3 mm^3^). MNI: Montreal Neurological Institute, corr: corrected, PFC: prefrontal cortex, OFC: orbitofrontal cortex, FWE: family-wise error. All labels are taken from the automatic anatomical labeling atlas. The significance threshold was set to *P* < 0.05 (FWE corrected). ^*^small volume corrected; all other activations are sig. at whole-brain level.

### Neural correlates of affective picture processing

As expected, pictures that were a priori classified as negative were rated as significantly more negative (mean = 1.6, *s.d.* = 0.39) than those that had been classified as neutral (mean = 3.57, *s.d.* = 0.42; *F*_(1,130)_ = 1033.02, *P* < 0.001; Table 2). The experimental manipulation (stress *vs* control) and the *CNR1* genotype had no influence on these emotionality ratings (all *F* ≤ 0.77, all *P* ≥ 0.381). With respect to reaction times, participants were faster to respond to neutral than negative pictures (*F*_(1,129)_ = 5.28, *P* = 0.023), which is in line with previous studies showing that emotional stimuli automatically capture our attention and are, for example, viewed longer than neutral pictures (Hajcak *et al.*, 2010), which might indicate more in-depth processing of negative material as is supported by electroencephalography studies with a high temporal sensitivity (Palomba *et al.*, 1997; Hajcak *et al.*, 2010). In addition, GG genotype carriers rated the pictures, irrespective of picture emotionality, faster than AA/AG genotype carriers (*F*_(1,129)_ = 4.04, *P* = 0.047).

In line with previous findings (van Stegeren, 2009; van Stegeren *et al.*, 2010; Etkin *et al.*, 2011), negative (*vs* neutral) picture processing led overall to significant increases in activation in brain regions associated with affective processing and emotion regulation. Specifically, irrespective of stress and *CNR1* variant, the presentation of negative pictures increased bilateral activation of the amygdala (right: *t* = 10.81, *P*_FWE_ < 0.001, *k* = 84; left: *t* = 10.68, *P*_FWE_ < 0.001, *k* = 64), vlPFC (right: *t* = 6.01, *P*_FWE_ < 0.001, *k* = 36, left: *t* = 4.74, *P*_FWE_ < 0.001, *k* = 46), vmPFC (right: *t* = 6.07, *P*_FWE_ < 0.001, *k* = 114; left: *t* = 6.31, *P*_FWE_ < 0.001, *k* = 138) and insula (right: *t* = 8.78, *P*_FWE_ < 0.001, *k* = 122), with the left insula even surviving FWE correction at whole-brain level (left: *t* = 11.59, *P*_FWE_ < 0.001, *k* = 2,053). In addition, the occipital inferior gyrus (left: *t* = 12.33, *P*_FWE_ < 0.001, *k* = 1,242) and anterior parietal regions [post-central gyrus (right: *t* = 17.74, *P*_FWE_ < 0.001, *k* = 1,226), supramarginal gyrus (left: *t* = 6.15, *P*_FWE_ < 0.001, *k* = 20)], which have been associated with emotional arousal and the regulation of an individual’s internal state (`as-if-body-loop’; Damasio *et al.*, 2000; Anders *et al.*, 2004), were more strongly activated when negative pictures were presented (Table 3). No brain regions were stronger activated during neutral compared to negative picture presentation.

### *CNR1* genotype modulates prefrontal activity and connectivity with the amygdala during affective picture processing

**Fig. 2:**
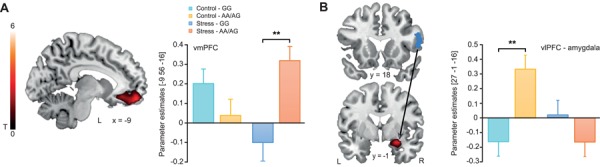
Stress and *CNR1* genotype effects on brain activity during affective picture processing. (**A**) Activity in the vmPFC was increased in rs1049353 AA/AG compared to GG genotype carriers for negative (*vs* neutral) picture processing under stress. (**B**) Under no-stress control conditions, the vlPFC showed enhanced functional connectivity with the amygdala when AA/AG compared to GG genotype carriers viewed negative (*vs* neutral) pictures. Activations are superimposed on coronal sections of a T1-weighted template image and represented in red. vmPFC: ventromedial prefrontal cortex, vlPFC: ventrolateral prefrontal cortex, L corresponds to the left, R to the right side of the brain and error bars represent SEM. ^**^P < 0.01

Corroborating earlier studies that emphasized an essential role of eCBs in the maintenance of emotional homeostasis in the face of a stressor (Lutz, 2009; McLaughlin *et al.*, 2014), the rs1049353 SNP modulated brain activation in response to negative (*vs* neutral) pictures under stress. Specifically, we observed an interaction between treatment (stress *vs* control) and *CNR1* genotype (rs1049353 AA/AG *vs* GG genotype) on vmPFC activity for negative *vs* neutral pictures (left: *t* = 3.55, *P*_FWE_ = 0.034, *k* = 24; Figure 2A). Post-hoc tests revealed that under stress, AA/AG compared to GG genotype carriers showed enhanced activity of the vmPFC (left: *t* = 3.64, *P*_FWE_ = 0.035, *k* = 22), whereas there were no genotype-dependent effects in the control condition. No main effects of *CNR1* genotype or treatment were observed, nor were any other brain areas significantly modulated by *CNR1* genotype or treatment (no suprathreshold clusters).

In order to gain insight into the network structure underlying the modulatory effect of the *CNR1* genotype on stress-induced changes in neural affective processing, we performed, in a next step, functional connectivity analyses. These analyses revealed a significant *CNR1* genotype × treatment interaction for the coupling of the vlPFC and the amygdala (*t* = 3.43, *P*_FWE_ = 0.013, *k* = 27). As displayed in Figure 2B, AA/AG compared to GG genotype carriers showed significantly increased vlPFC-amygdala connectivity under no-stress control conditions during negative picture processing (*t* = 3.76, *P*_FWE_ = 0.007, *k* = 38), whereas genotype groups did not differ after stress (no suprathreshold clusters).

### Memory performance in AA/AG genotype carriers correlates with activation of and connectivity between limbic areas after stress

Performance in the surprise free recall test 24 h after picture presentation was overall rather moderate (participants recalled on average 20 ± 8% of all pictures). As expected, memory was significantly better for negative (mean = 6, *s.d.* = 2) than for neutral pictures (mean = 3, *s.d.* = 2; *F*_(1,130)_ = 203.98, *P* < 0.001; Table 2). Performance in the recognition task was overall very high, with an average hit rate of 88% and a false alarm rate of only 15%. In line with the free recall data, we observed superior recognition memory for negative items (increased hit rate, reduced false alarm rate; all *F*_(1,129)_ ≥ 25.46, all *P* < 0.001; Table 2). An increased sensitivity index d′ (*F*_(1,129)_ = 140.53, *P* < 0.001) and higher confidence ratings (*F*_(1,129)_ = 99.68, *P* < 0.001) for negative relative to neutral pictures lent further support for the emotional memory enhancement (Table 2). Importantly, recall and recognition performance for negative and neutral pictures as well as confidence ratings were unaffected by stress and *CNR1* genotype (all *F* ≤ 2.49, all *P* ≥ 0.117).

**Fig. 3:**
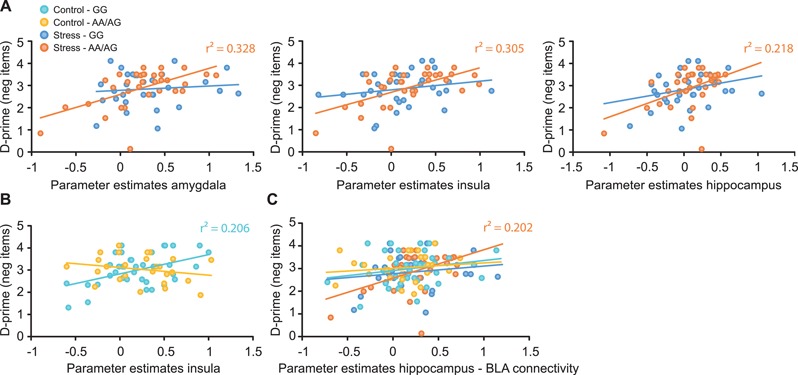
Correlations between brain activity during encoding of negative (*vs* neutral) pictures and emotional memory performance, expressed as sensitivity index d′ for negative pictures. (**A**) Following the stress manipulation, activity of the amygdala, insula and hippocampus positively correlated with memory performance in stressed AA/AG genotype carriers. (**B**) In the no-stress control condition, insula activity positively correlated with memory performance in GG genotype allele carriers. (**C**) Enhanced functional connecitivity of the hippocampus with the BLA during negative (*vs* neutral) picture encoding was associated with enhanced memory performance for negative pictures only in stressed AA/AG genotype carriers.

In order to investigate whether the neural underpinnings of emotional memory formation were affected by stress and the *CNR1* polymorphism, we correlated brain activity for negative compared to neutral items during encoding with the 24 hour-delayed memory performance for negative pictures. Since free recall performance was rather moderate and variance was small, we used the sensitivity index d′ in our correlation analyses. In line with a crucial role of limbic brain regions in the processing of and memory formation for emotional material (McGaugh, 2004; LaBar and Cabeza, 2006), our analyses revealed overall significant clusters in the amygdala (left: *t* = 2.85, *P*_FWE_ = 0.046, *k* = 10), insula (right: *t* = 4.24, *P*_FWE_ = 0.001, *k* = 113; left: *t* = 4.52, *P*_FWE_ = 0.001, *k* = 90) and hippocampus (left: *t* = 3.76, *P*_FWE_ = 0.008, *k* = 36) during negative compared to neutral picture presentation. These clusters were positively correlated with participants’ memory performance for negative pictures (amygdala: left: *r* = 0.310, *P* < 0.001; insula: right: *r* = 0.310, *P* = 0.001, left: *r* = 0.275, *P* = 0.001; hippocampus: *r* = 0.209, *P* = 0.016). Importantly, the neural correlates of emotional memory enhancement were modulated by stress and *CNR1* genotype. Specifically, we observed that in stressed AA/AG genotype carriers emotional memory performance positively correlated with clusters in the amygdala (left: *t* = 4.24, *P*_FWE_ = 0.003, *k* = 28; *r* = 0.571, *P* = 0.001), insula (left: *t* = 4.13, *P*_FWE_ = 0.012, *k* = 62; *r* = 0.555, *P* = 0.001, right: *t* = 3.61, *P*_FWE_ = 0.039, *k* = 26; *r* = 0.571, *P* = 0.001) and hippocampus (left: *t* = 4.16, *P*_FWE_ = 0.009, *k* = 56; *r* = 0.468, *P* = 0.006; Figure 3A). In contrast, no such correlations were found in stressed GG genotype carriers (no suprathreshold clusters, all *r* ≤ 0.289, *P* ≥ 0.108). In the control condition, however, there were no significant clusters that were activated during negative picture encoding and that correlated with emotional memory performance in AA/AG genotype carriers (no suprathreshold clusters, all *r* ≤ 0.247, *P* ≥ 0.215), whereas in GG genotype carriers emotional memory performance was positively correlated with activation of the insula (right: *t* = 4.22, *P*_FWE_ = 0.008, *k* = 14; *r* = 0.455, *P* = 0.004; Figure 3B). After controlling for multiple comparisons by dividing α by the number of correlations calculated (0.05 / 4), all clusters and correlations but the cluster in the right insula remained significant at a corrected *α* threshold of *P* < 0.0125. In support of *CNR1* genotype-dependent differences in the neural basis of emotional memory formation, the correlations between emotional memory performance and activation of limbic brain regions during negative compared to neutral picture encoding significantly differed between AA/AG and GG genotype carriers in the stress (amygdala and insula: both *z* between −2.06 and −1.61, *P* ≤ 0.054) and control condition (insula: *z* = 2.72, *P* = 0.003).

In addition to the activation of single brain regions, we analyzed how functional connectivity patterns during negative picture encoding may relate to later memory performance. Interestingly, functional connectivity of the hippocampus and the BLA was associated with enhanced emotional memory only in stressed AA/AG genotype carriers (*t* = 3.78, *P*_FWE_ = 0.004, *k* = 31; *r* = 0.449, *P* = 0.009; Figure 3C), an effect that survived the adapted threshold of *P* < 0.0125 to control for multiple comparisons. No such correlations were observed in stressed GG genotype carriers or AA/AG and GG genotype carriers in the control condition (no suprathreshold clusters, all *r* between −0.112 and 0.213, all *P* > 0.102; no differences between these groups: all *z* ≥ −1.26, all *P* ≥ 0.103).

## Discussion

The eCB system has been suggested to act as an emotional buffer under stress (Morena and Campolongo, 2014). However, how eCBs may modulate affective responding in humans remained unclear. Here we used an imaging genetics approach to investigate how a variant of the gene coding for the CB1 receptor may alter the neural processing of affective information under stress. Our results show, in line with a crucial role of eCB signaling in affective responding, significant changes of affective processing under stress depending on the CB1 receptor gene variant.

More specifically, we obtained stronger activity of the vmPFC in AA/AG compared to GG genotype carriers when processing emotionally negative (*vs* neutral) information after stress. The mPFC coordinates cognitive, emotional and behavioral responses to stressful stimuli and regulates glucocorticoid-mediated negative feedback inhibition of the HPA axis (Diorio *et al.*, 1993; McLaughlin *et al.*, 2014). The vmPFC specifically is known to play a crucial role in emotion regulation and extinction processes (Ochsner and Gross, 2005; Kalisch *et al.*, 2006; Goldin *et al.*, 2008) and reduced activation of the vmPFC during negative picture processing has been associated with depression (Brassen *et al.*, 2008), which suggests that the stronger recruitment of the vmPFC may allow more efficient affective processing and emotion regulation under stress. Indeed, the vmPFC has been shown to regulate limbic brain regions that are involved in the generation of emotional responses (Etkin *et al.*, 2011) and enhanced activation of this brain region is associated with reduced negative affect (Urry *et al.*, 2006). ECB signaling appears to be crucial for effective mPFC functioning (Morena *et al.*, 2016). Specifically, eCBs have been shown to regulate GABAergic inhibition of the mPFC (McLaughlin *et al.*, 2014) and increased eCB signaling in the mPFC and amygdala were able to suppress anxiety (Rubino *et al.*, 2008). The eCB-induced increase in dopamine and the reduced GABAergic inhibition of the mPFC (Chiu *et al.*, 2010) in concert with enhanced serotonergic activation (McLaughlin *et al.*, 2012) may promote self-focused emotion regulation and active stress coping strategies dependent on the mPFC (Ochsner *et al.*, 2004; McLaughlin *et al.*, 2014), which is in line with anxiety-reducing and antidepressant-like effects of CB1 receptor agonists (Bambico *et al.*, 2007; Akirav, 2011). These mechanisms may contribute to the proposed protective effect of the rs1049353 AA/AG genotype against stress-related psychopathologies.

Interestingly, we obtained under no-stress control conditions differential prefrontal engagement during affective processing in AA/AG compared to GG genotype carriers. In particular, the vlPFC, another critical area for emotion regulation (Ochsner and Gross, 2005), showed in the control condition stronger connectivity with the amygdala during affective processing in AA/AG compared to GG genotype carriers. Although functional connectivity data do not allow conclusions regarding the direction of the interaction, this finding is generally in line with previous studies suggesting that the vlPFC inhibits activation of the amygdala, thereby diminishing the influence of the amygdala during affective processing, which is crucial for successful emotion regulation in the face of threatening stimuli (Banks *et al.*, 2007; Wager *et al.*, 2008; Etkin *et al.*, 2011). Thus, this increased vlPFC–amygdala connectivity may represent another mechanism contributing to beneficial effects of the AA/AG genotype in coping with adverse events. While these neural data provide evidence for differences between AA/AG and GG genotype carriers in affective processing under stress and control conditions, it is important to note that these differences at the neural level were not accompanied by behavioral changes (i.e. changes in emotionality ratings), which may be, at least in part, due to the reduced sensitivity of the four-point rating scale. Interestingly, however, GG genotype carriers were generally faster in their emotionality ratings, which might be indicative of more automatic, reflexive affective responding.

Beyond a mere modulation of affective processing under stress, the eCB system has been implicated in memory formation for emotional events, most likely through its influence on rapid glucocorticoid signaling (Campolongo *et al.*, 2009; Atsak *et al.*, 2012a; Atsak *et al.*, 2012b; Atsak *et al.*, 2015). In line with a number of previous studies (for a review see Hamann, 2001), our results showed better memory for negative compared to neutral information. This emotional memory enhancement is commonly assumed to rely on the actions of catecholamines and glucocorticoids in the amygdala, which then modulate memory processes in areas such as the hippocampus (McGaugh, 2000; LaBar and Cabeza, 2006; Roozendaal *et al.*, 2009). In line with these ideas, we obtained, across genotype and treatment groups, significant correlations between memory performance for emotionally arousing stimuli and activity in the amygdala, insula and hippocampus. The neural underpinnings of the emotional memory enhancement, however, were distinct in carriers of the AA/AG and GG genotype of the *CNR1* gene polymorphism. When performing experimental group-dependent analyses, the correlations between hippocampal, amygdala and insula activity and emotional memory performance were observed in stressed AA/AG but not GG genotype carriers. Moreover, we obtained a significant correlation between the functional connectivity of the BLA and hippocampus after stress, as predicted by prominent models of emotional memory formation (Roozendaal *et al.*, 2004; Roozendaal and McGaugh, 2011; Roozendaal and Hermans, 2017), in stressed AA/AG but not GG genotype carriers. This result is generally in line with the finding that injection of a CB1 receptor agonist into the BLA improved emotional memory in rats, whereas the corticosterone-induced emotional memory enhancement was blocked already by injection of a very low dose of a CB1 receptor antagonist into the BLA (Campolongo *et al.*, 2009). These findings support the view that a stress-induced increase in glucocorticoids stimulates eCB signaling, leading to increased (noradrenergic) activity in the BLA, most likely by inhibiting GABAergic influences (Duvarci and Pare, 2007; Hill and McEwen, 2009), which then enhances memory consolidation through changes in hippocampal synaptic plasticity. Similarly to the data on affective processing, however, it is important to note that the neural differences in memory formation did not translate into performance differences in the present study. The absence of behavioral differences may be owing to the overall rather moderate performance level in the surprise memory tests. Alternatively, differential memory performance might be revealed in more sophisticated memory tests that assess the actual level of elaboration of the encoded material (Schwabe and Wolf, 2013). CB1 genotype-related changes in the neural signature of emotional memory formation may result in encoding and consolidation changes that are highly relevant in the context of PTSD and anxiety disorders and are further related to the protective effects of the AA/AG genotype of the rs1049353 polymorphism on mental health.

The fact that we see a (potentially) beneficial influence of the rs1049353 AA/AG genotype on the neural correlates of affective processing and emotional memory enhancement under stress, but not under control conditions, is in line with some previous results suggesting that a certain degree of emotional arousal is needed for a modulation by eCB signaling (Campolongo *et al.*, 2012). Although it is unknown whether the rs1049353 polymorphism is functional or not, it has been postulated to affect mRNA stability (Chakrabarti *et al.*, 2006; Domschke *et al.*, 2008; Hill and Patel, 2013). Clinical studies showed rather mixed results so far. Specifically, AA/AG genotype carriers with high levels of childhood physical abuse reported greater avoidance and re-experiencing symptoms of the PTSD checklist (Mota *et al.*, 2015). However, some level of protection against stress and the development of depression in AA/AG genotype carriers has been demonstrated (Domschke *et al.*, 2008; Agrawal *et al.*, 2012), whereas in another study the increased risk for depression in participants with a history of abuse was not modulated by rs1049353 genotype. In addition, a neuroimaging study in healthy participants revealed diminished activation of the striatum in response to happy faces in GG genotype carriers, which may be indicative of reduced social reward responsivity (Chakrabarti *et al.*, 2006). This, however, may be dependent on the participants’ developmental phase, since another study did not observe such an effect in adolescent participants (Ewald *et al.*, 2016). In this study, adolescent AA/AG *vs* GG genotype carriers showed earlier recognition of fear and sadness when the facial expression, from which they were morphed, displayed anger (Ewald *et al.*, 2016). This effect was accompanied by increased amygdala and insula activation in response to angry faces *vs* non-facial control pictures. In line with a reduced amygdala response to happy faces in depressed G allele carriers (Domschke *et al.*, 2008), these findings may be interpreted as an increased warning signal against possible threats, serving a protective role (Ewald *et al.*, 2016). Based on these results and the proposal that the rs1049353 AA/AG genotype may result in a more stable mRNA (Hill and Patel, 2013), it is tempting to speculate that the AA genotype is associated with enhanced CB1 receptor functioning *in vivo*. Whereas future molecular studies are needed to explicitly test this prediction and further investigations are required to elucidate the rather mixed clinical results, we propose that enhanced CB1 receptor functioning in AA/AG genotype carriers improves emotion regulation strategies in the face of stress and perhaps the incorporation of emotional events into autobiographical memory, for which the hippocampus is essential (Cabeza and St. Jacques, 2007). In contrast to previous studies that suggested a role of eCBs and the CB1 receptor in the regulation of the HPA axis (McLaughlin *et al.*, 2014), we obtained no influence of the CB1 receptor polymorphism on the cortisol response to stress. The absence of such an effect suggests that the effects of the rs1049353 polymorphism on the neural underpinnings of affective processing and emotional memory formation are not simply driven by changes in the cortisol response to stress. It is very likely that the influence of eCB signaling on other neurotransmitter systems is crucial, such as the influence of eCBs on GABAergic neurons in the mPFC (McLaughlin *et al.*, 2014) and BLA (Duvarci and Paré, 2007), as well as on serotonergic pathways to other limbic brain regions (McLaughlin *et al.*^2012^). Additionally, eCB signaling is influenced by corticotropin-releasing factor, which is known to increased FAAH secretion, leading to augmented degradation of anandamide, which reduces its inhibitory tone on HPA axis activity (McLaughlin *et al.*, 2014; Morena *et al.*, 2016). Interestingly, BLA habituation in response to emotional faces, an indicator of fear extinction, was the most reduced in participants carrying genetic polymorphisms associated with enhanced CRF and FAAH signaling, an effect which mediated increased risk for anxiety disorders (Demers *et al.*, 2016). This finding suggests that although enhanced anandamide signaling may decrease anxiety, combined increases in CRF signaling may have somewhat paradoxical effects. Thereby, this study also highlights the importance of investigating gene × gene interactions and conducting pharmacogenetics studies before FAAH inhibitors as potential treatment options can be considered.

Although interactions between stress and genetic variants of the eCB system can be highly informative, in particular in the absence of direct pharmacological manipulations in humans, and may have a great potential for advancing our understanding of stress-related psychopathologies, it is important to note that effect sizes of single genetic polymorphisms are likely small (Dick *et al.*, 2015). Even for neural phenotypes, this seems to be the case (Franke *et al.*, 2016). Thus, our results need to be interpreted with caution and—given that the power to detect a small effect in single genetic variants is approximately 15%—require independent replication in highly powered studies (Duncan and Keller, 2011). Additionally, future studies could benefit from more sophisticated methods such as multilocus genetic profile or risk scores, which have successfully been used to investigate genetic differences in episodic memory (de Quervain and Papassotiropoulos, 2006) as well as in HPA axis functioning in interaction with early life stress (Pagliaccio *et al.*, 2014; Di Iorio *et al.*, 2017).

In sum, our results show that a variant of the gene coding for the CB1 receptor (rs10493453 AA/AG genotype) is associated with increased recruitment of prefrontal regions that are important for emotion regulation during affective processing under stress and with enhanced connectivity of the hippocampus with the BLA during emotional memory formation. These data may point to improved emotion regulation abilities and more appropriate consolidation of emotional events into autobiographical memory in individuals with one or more copies of the minor A allele of this polymorphism. This modulation of affective and cognitive processing under stress may contribute to a certain degree of protection against stress-related disorders such as PTSD. Indeed, first evidence suggests that the eCB system may be an effective target for treating the cognitive and affective characteristics of PTSD (Hill *et al.*, 2006; Ganon-Elazar and Akirav, 2012; Akirav, 2013; Trezza and Campolongo, 2013; Korem *et al.*, 2016). Specifically, in rats it was shown that injections of a CB1/CB2 receptor agonist into the BLA and hippocampus prevented the stress-induced glucocorticoid receptor upregulation in these brain regions as well as in the PFC and prevented an impairment in fear extinction (Ganon-Elazar and Akirav, 2013). In combination with evidence suggesting glucocorticoid-based therapies for the attenuation of aversive memories (de Quervain *et al.*, 2017), these findings may hopefully advance the development of new treatment options for PTSD and other stress-related disorders that are characterized by aberrant affective processing.

## Supplementary Material

Supplementary Data
