# Supplementary material for: An endocannabinoid receptor polymorphism modulates affective processing under stress
Source: Soc Cogn Affect Neurosci. 2018 Oct 2;13(11):1177–89. doi: 10.1093/scan/nsy083 (PMC6234318; doi:10.1093/scan/nsy083)
Supplement: Supplementary Data [file nsy083_suppl.docx]

**Supplementary results**

Given that the genotypic distribution nearly violated Hardy-Weinberg equilibrium and in light of different homozygous A allele frequencies reported in previous studies (Domschke *et al.*, 2008; Agrawal *et al.*, 2012), we reanalyzed our data without AA genotype carriers. Since all AA genotype carriers were in the stress condition, we refrained from repeating the post-hoc analyses in the control group. Importantly, we observed that our results remained largely unchanged.

**Successful stress induction by the TSST**

In line with our previous analyses, genotype did neither alone nor in interaction with treatment influence systolic blood pressure, cortisol concentrations or TSST ratings (all main and interaction effects: all *F* ≤ 1.81, all *P* ≥ 0.181). As before, stress led to increases in blood pressure, cortisol, negative mood and restlessness (all time × treatment effects: all *F* ≥ 16.88, all *P* < 0.001), and the treatment was rated as more difficult, unpleasant and stressful following the TSST (all *F* ≥ 73.01, all *P* < 0.001). Similarly unchanged, a time × treatment × genotype interaction for diastolic blood pressure (*F*_(3,129)_ ≥ 3.20, *P =* 0.026) showed that in rs1049353 GG genotype carriers, diastolic blood pressure increased during and immediately following the stress induction (both *F*_(1,72)_ ≥ 7.57, both *P* ≤ 0.008), whereas in AA/AG genotype carriers no such effect was observed (both *F*_(1,55)_ ≤ 1.11, both *P* ≥ 0.297). Whereas GG genotype carriers were overall more restless than AG genotype carriers (*F*_(1,126)_ = 5.24, *P* = 0.024), negative mood was not affected by genotype (all main and interaction effects: all *F* ≤ 0.63, all *P* ≥ 0.429). Independent of stress and genotype, participants became increasingly tired during the course of the experiment (time: *F*_(2,125)_ = 92.15, *P* < 0.001). Thus, results of subjective and physiological stress measures remained unchanged when AA genotype carriers were excluded.

***CNR1* genotype modulates neural correlates of affective picture processing**

Similarly unchanged were the results regarding the emotionality ratings, reaction times and the neural correlates of affective processing. Whereas negative pictures were rated significantly more negative (mean = 1.6, SD = 0.35) than neutral pictures (mean = 3.6, SD = 0.39; *F*_(1,126)_ = 969.32, *P* < 0.001), these ratings remained unaffected by treatment and *CNR1* genotype (all *F* ≤ 1.24, all *P* ≥ 0.267). Reaction times were faster for neutral compared to negative pictures (*F*_(1,125)_ = 5.52, *P* = 0.020) and GG genotype carriers, independent of picture emotionality, were faster than AG carriers in their emotionality ratings (*F*_(1,125)_ = 5.99, *P* = 0.016). Regarding the neural correlates of affective picture processing, a significant treatment (stress vs. control) × *CNR1* genotype (rs1049353 AA/AG vs. GG genotype) interaction on vmPFC activity for negative vs. neutral pictures (left: *t* = 3.62, *P*_FWE_ = 0.029, *k* = 33) revealed that under stress, AG compared to GG genotype carriers showed enhanced vmPFC activity (left: *t* = 3.83, *P*_FWE_ = 0.022, *k* = 45), whereas no genotype-dependent effects were shown in the control condition. Results of our functional connectivity analyses were confirmed and revealed a significant *CNR1* genotype × treatment interaction for the coupling of the vlPFC and two clusters in the amygdala (cluster 1: *t* = 3.32, *P*_FWE_ = 0.018, cluster 2: *t* = 3.30, *P*_FWE_ = 0.019, both *k* = 9). In accordance with our previous analyses (no AA genotype carriers were present in the control condition) AG compared to GG genotype carriers in the no-stress conditions showed enhanced vlPFC-amygdala connectivity (*t* = 3.76, *P*_FWE_ = 0.007, *k* = 38), whereas genotype groups did not differ in the stress condition (no suprathreshold clusters).

**Memory performance in AA/AG genotype carriers correlates with activation of and connectivity between limbic areas after stress**

Free recall and recognition performance for negative and neutral pictures, as well as confidence ratings remained unaffected by stress and *CNR1* genotype (all *F* ≤ 2.54, all *P* ≥ 0.114). Our analyses correlating memory performance (d’) for negative items with brain activation and connectivity during negative vs. neutral picture encoding showed similar results as before. Overall, significant clusters were observed in the insula (right: *t* = 4.38, *P*_FWE_ = 0.001, *k* = 113; left: *t* = 4.41, *P*_FWE_ = 0.001, *k* = 87) and hippocampus (left: *t* = 3.68, *P*_FWE_ = 0.011, *k* = 38) during negative compared to neutral picture presentation, but no longer in the amygdala (no suprathreshold clusters). These clusters positively correlated with participants’ memory performance for negative pictures (insula: right: *r* = 0.316, *P* < 0.001, left: *r* = 0.283, *P* = 0.001; hippocampus: *r* = 0.223, *P* = 0.012). Looking at these correlations in our experimental groups, we observed that in stressed AG genotype carriers emotional memory performance positively correlated with clusters in the amygdala (left: *t* = 4.37, *P*_FWE_ = 0.003, *k* = 35; *r* = 0.614, *P* < 0.001), insula (left: *t* = 4.41, *P*_FWE_ = 0.007, *k* = 97; *r* = 0.595, *P* = 0.001, right: *t* = 3.59, *P*_FWE_ = 0.043, *k* = 39; *r* = 0.614, *P* < 0.001) and hippocampus (left: *t* = 4.02, *P*_FWE_ = 0.016, *k* = 79; *r* = 0.483, *P* = 0.008). Controlling for multiple comparisons, all clusters and correlations but the cluster in the right insula and left hippocampus remained significant at a corrected *α* threshold of *P* < 0.0125. The correlations between emotional memory and clusters in the amygdala and insula significantly differed between AG and GG genotype carriers in the stress condition (left amygdala: z = 2.32, *P* = 0.013; left insula: z = 1.78, *P* = 0.038; trend right insula: z = 1.60, *P* = 0.055). In addition, functional connectivity of the hippocampus and the BLA was associated with enhanced emotional memory only in stressed AG genotype carriers (*t* = 3.90, *P*_FWE_ = 0.003, *k* = 39; *r* = 0.472, *P* = 0.010), an effect that survived the adapted threshold of *P* < 0.0125 to control for multiple comparisons. No such correlations were observed in stressed GG genotype carriers or AG and GG genotype carriers in the control condition (no suprathreshold clusters, all *r* < 0.222, all *P* > 0.181; no differences between these groups: all *z* ≥ 1.274, all *P* ≥ 0.101). Genotype groups in the control condition did not change (no AA genotype carriers in the control condition) and results thus remained unchanged.

**References**

Agrawal, A., Nelson, E. C., Littlefield, A. K., Bucholz, K. K., Degenhardt, L., Henders, A. K., Madden, P. A. F., Martin, N. G., Montgomery, G. W., Pergadia, M. L., Sher, K. J., Heath, A. C. & Lynskey, M. T. (2012) 'Cannabinoid receptor genotype moderation of the effects of childhood physical abuse on anhedonia and depression', *Arch Gen Psychiatry*, **60**(7), pp. 732-740.

Domschke, K., Dannlowski, U., Ohrmann, P., Lawford, B., Bauer, J., Kugel, H., Heindel, W., Young, R., Morris, P., Arolt, V., Deckert, J., Suslow, T. & Baune, B. T. (2008) 'Cannabinoid receptor 1 (CNR1) gene: impact on antidepressant treatment response and emotion processing in major depression', *Eur Neuropsychopharmacol*, **18**(10), pp. 751-759.
